# FET proteins regulate lifespan and neuronal integrity

**DOI:** 10.1038/srep25159

**Published:** 2016-04-27

**Authors:** Martine Therrien, Guy A. Rouleau, Patrick A. Dion, J. Alex Parker

**Affiliations:** 1 CHUM Research Center, Montreal, H2X 3H8, Canada; 2 Pathology and Cell biology department, University of Montreal, Montreal, H3T 1J4, Canada; 3 Neurology and Neurosurgery department, McGill University, Montreal, H3A 0G4, Canada; 4 Montreal Neurological Hospital, Montreal, H3A 2B4, Canada; 5 Department of Neuroscience, University of Montreal, Montreal, H3T 1J4, Canada

## Abstract

The FET protein family includes FUS, EWS and TAF15 proteins, all of which have been linked to amyotrophic lateral sclerosis, a fatal neurodegenerative disease affecting motor neurons. Here, we show that a reduction of FET proteins in the nematode *Caenorhabditis elegans* causes synaptic dysfunction accompanied by impaired motor phenotypes. FET proteins are also involved in the regulation of lifespan and stress resistance, acting partially through the insulin/IGF-signalling pathway. We propose that FET proteins are involved in the maintenance of lifespan, cellular stress resistance and neuronal integrity.

Mutations in fused-in-sarcoma (*FUS*) are one of the causes of amyotrophic lateral sclerosis (ALS)^1^,^2^,^3^, a fatal neurodegenerative disease causing loss of motor neurons. Mutations are found in different domains of the protein and cause its cellular mislocalization^3^. The presence of FUS in the cytoplasm suggests a gain of toxic function mechanism, but the depletion of FUS from the nucleus also points to a loss of normal function being implicated in motor neuron degeneration. Due to its nature as an RNA/DNA binding protein, FUS has been shown to participate in many cellular functions including translation, splicing, and RNA transport^4^. FUS is part of the FET protein family that includes two other RNA binding proteins; Ewing sarcoma breakpoint region 1 (*EWSR1* gene encoding the EWS protein) and TBP associated factor 15 (*TAF15*). These proteins are highly similar and it is thought that they share common functions^5^,^6^. Furthermore, *EWSR1* and *TAF15* mutations have been linked to some sporadic cases of ALS^7^,^8^. However, how mutant FET proteins cause neuronal loss is still unclear.

Many proteins associated with ALS are evolutionarily conserved in the nematode *Caenorhabditis elegans. C*. *elegans* are transparent nematodes that have been used to make important contributions to the fields of neuroscience and aging. More recently *C. elegans* has emerged as a useful model to study human diseases, namely conserved aspects of age-dependent neurodegeneration^9^.

To better understand the function of FET proteins, we characterised the *fust-1(tm4439)* deletion mutant in worms. *C. elegans* has a simple, largely non-redundant genome and many highly conserved human genes have a single orthologue in the nematode. Here, *fust-1* is the orthologue of *FUS*, *EWSR1* and *TAF15*. Using a loss of function mutation, we show that *fust-1* is a key gene acting to regulate neuronal integrity, lifespan and cellular stress responses. Also, for some of these functions, *fust-1* is an active component of the insulin/IGF-like signalling pathway (ISS).

## Results

### FUST-1 is required for neuronal integrity

**Figure 1:**
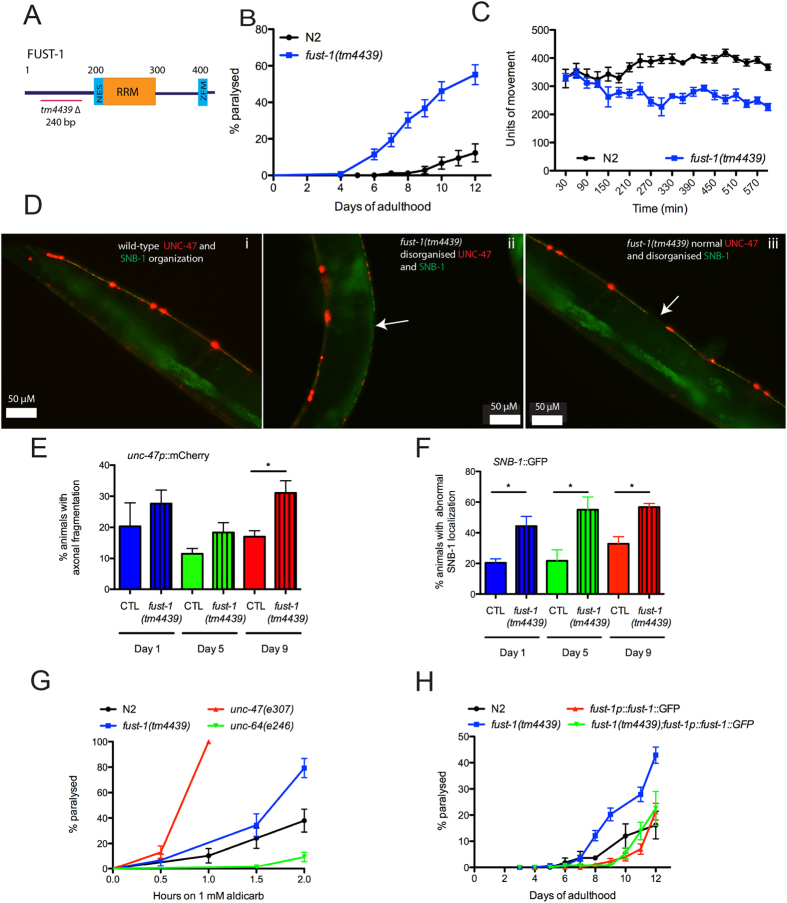
Deletion of *fust-1* causes motility impairment and loss of neuronal integrity. (**A**) *C. elegans fust-1* is the ortholog of human *FUS*, *EWSR1* and *TAF15* and contains the conserved RNA recognition motif (RRM), nuclear export signal (NES) and zinc finger motif (ZFM). *fust-1(tm4439)* is a 240 bp deletion. **(B–C**) Loss of *fust-1* expression causes age-dependant paralysis **(B)** on solid media (p value < 0.0001) and **(C)** the phenomenon is accelerated in liquid media (p value < 0.0001) **(D–F) (D)** *unc-47* promoter (red) and SNB-1 (green) were used to visualise motor neurons and synapse formation. Shown is the normal expression pattern in wild type animals (i), while decreased expression of *fust-1* caused gaps along the motor neurons (ii), and disorganisation of the SNB-1 protein (ii-iii). Quantification of the number of animals at day 1, 5 and 9 with **(E)** axonal fragmentation in motor neuron (*unc-47p*::mCherry) (*p value < 0.05, n ≥ 100 for each condition) and **(F)** synaptic disorganisation (*unc-25p*::SNB-1::GFP) (*p value < 0.05, n ≥ 100 for each condition). **(G)** *fust-1(tm4439)* mutants are more sensitive to aldicarb than N2 and *unc-64(e246)* but less than the hypersensitive strain *unc-47(e307)* p < 0.001. **(H**) Overexpression of *fust-1* rescues the age dependant paralysis phenotype observed in *fust-1(tm4439)* mutants (p value < 0.001).

To understand the function and role of the FET proteins, we characterised *fust-1*, which encodes the *C. elegans* orthologue of *FUS, EWSR1, TAF15*. At the protein level, FUST-1 shares 50% identity with the FUS human protein, 32% identity with EWS and 35% identity with TAF15 (Supplementary Material, Fig. S1A). Bioinformatic analyses using NetNes (http://www.cbs.dtu.dk/services/NetNES/) and Prosite (http://prosite.expasy.org/scanprosite/) confirmed the conservation of the main functional domains of FET proteins including the RNA binding domain, the zinc finger motif and the nuclear export signal (Fig. 1A and Supplementary Material, Figs S1 and S2A,B). To investigate the role of FUST-1, we used a *C. elegans* deletion mutant strain, *fust-1(tm4439)* which contains a homozygous 240 base pair deletion in the N terminal part of the gene. The *tm4439* allele is an in-frame deletion spanning 15 bp of the second intron, and 225 bp of exon 3 that is predicted to code for a protein product missing 75 amino acids compared to wild type FUST-1. While *fust-1* mRNA level increases during adulthood in wild type animals, *fust-1(tm4439)* mutants exhibit a similar expression level throughout adulthood which is at least 50% lower than the expression level of wild-type worms at day 1 (Supplementary Material, Fig. S2C). Thus, *fust-1(tm4439)* is likely a hypomorphic mutation that results in decreased RNA expression and may produce a defective protein product, suggesting that these worms could be used to model a partial loss of function of FUST-1.

Previous reports studying the function of the ortholog of *FUS* in *Drosophila, Cabeza*, have suggested that a decreased expression of *Cabeza* in flies induces neuronal dysfunction and defects in neuromuscular junction morphology^10^,^11^,^12^,^13^. To evaluate if this function was conserved in *C. elegans*, deletion mutant worms were evaluated for age-dependent paralysis, a motor phenotype that has been shown to be a good predictor of neuronal integrity^14^,^15^,^16^. At day 1 of adulthood, *fust-1(tm4439*) mutants showed normal motor behaviour when compared to wild-type N2 worms, but as the mutants aged they showed progressive motility defects leading to paralysis, reaching 66% paralysis by day 12 of adulthood compare to the 13% observed for wild-type N2 controls (Fig. 1B and Supplementary Material, Table S1A). We have previously shown that the paralysis phenotype that occurs when worms are grown on solid media after several days can be observed within hours when the worms are grown in liquid culture^17^,^18^. The swimming behaviour of *C. elegans* is an energetically costly activity that actively engages the neuromuscular junction and may be a phenotype relevant to the study of the health of motor neurons. To study the movement of the animals in liquid, worms were placed in a 96-well plate and their movements were evaluated with an automated method that measures locomotion activity based on infrared beam scattering^19^. With this method, *fust-1* mutants initially exhibit normal motility behaviour but show a drastic decrease in movement over time (Fig. 1C and Supplementary Material, Table S1B).

Next we assessed the integrity of GABAergic motor neurons using an *unc-47p*::mCherry reporter strain^20^. UNC-47 is a GABA vesicular transporter and is expressed in the 26 GABAergic motor neurons of the worm^21^. At day 9, *fust-1(tm4439)* mutants exhibit an increase in the number of gaps or breaks along the ventral cord probably due to axonal fragmentation (Fig. 1D,E) that coincides with the onset of the paralysis phenotype.

To evaluate potential synaptic dysfunction, an *unc25p::snb-1::GFP* reporter strain was used. SNB-1 is a synaptic vesicle protein and has been used to visualize synapses^22^. Starting at day 1 of adulthood, *fust-1* deletion mutant worms exhibit abnormal organization of SNB-1 protein compared to wild-type worms. SNB-1 abnormal localization affected motor neurons that also showed gaps along their axons (Fig. 1D ii) or could affect neurons prior to breakage of the axons (Fig. 1D iii). The proportion of worms with abnormal SNB-1 localization increased with aging reaching 60% of the worms at day 9 compared to 35% of the wild-type worms (Fig. 1F), however there was no change in the overall intensity of SNB-1 protein between the wild-type and *fust-1* mutant worms (Supplementary Material, Fig. S2D).

To evaluate the health of the neuromuscular junction, worms were exposed to aldicarb (2-methyl-2(methylthio) propanal o-[(methylamino)-carbonyl] oxime), an acetylcholine esterase inhibitor that causes the build-up of acetylcholine at the neuromuscular junction leading to paralysis^23^. Worms with defects in vesicular release at the neuromuscular junction showed hypersensitivity to aldicarb, similar to *unc-47* mutants^24^, or resistance, similar to *unc-64/Syntaxin* mutants^25^. *fust-1* mutants showed hypersensitivity to aldicarb when compared to wild-type N2, reaching 80% paralysis compared to 40% for the wild-type control after two hours in aldicarb (Fig. 1G). These data suggest an abnormal function of the neuromuscular junction perhaps due to a decrease of GABA release in the *fust-1* mutants.

To confirm that the effects observed in our *fust-1(tm4439)* worms were due to the loss of function of *fust-1*, we generated a transgenic worm expressing full-length *fust-1* linked to GFP under the control of its own promoter (*fust-1p::fust-1::GFP*). This strain displays GFP expression throughout development and adulthood with expression in the head, pharynx, intestine and tail of the adult worm (Supplementary Material, Fig. S3A). In some cells of the pharynx and the tail of the adult animal, FUST-1 was localized in the nucleus, similar to what is observed in humans (Supplementary Material, Fig. S3B). When crossed to the mutant worms, the *fust-1p::fust-1::GFP* construct completely rescued the paralysis phenotype of the *fust-1(tm4439*) worms (Fig. 1H and Supplementary Material, Table S1C) suggesting that indeed the motor phenotype is due to a loss of *fust-1*. Interestingly, the transgenic worms that overexpress *fust-1* do not exhibit motor impairment, suggesting that, in *C. elegans*, the overexpression of *fust-1* under the control of its own promoter is not intrinsically toxic.

Overall, these results suggest that synaptic dysfunction precedes neuronal loss and that aging could promote the development of the motor phenotype observed in the *fust-1(tm4439*) deletion mutant worms.

### FUST-1 is involved in lifespan regulation

**Figure 2:**
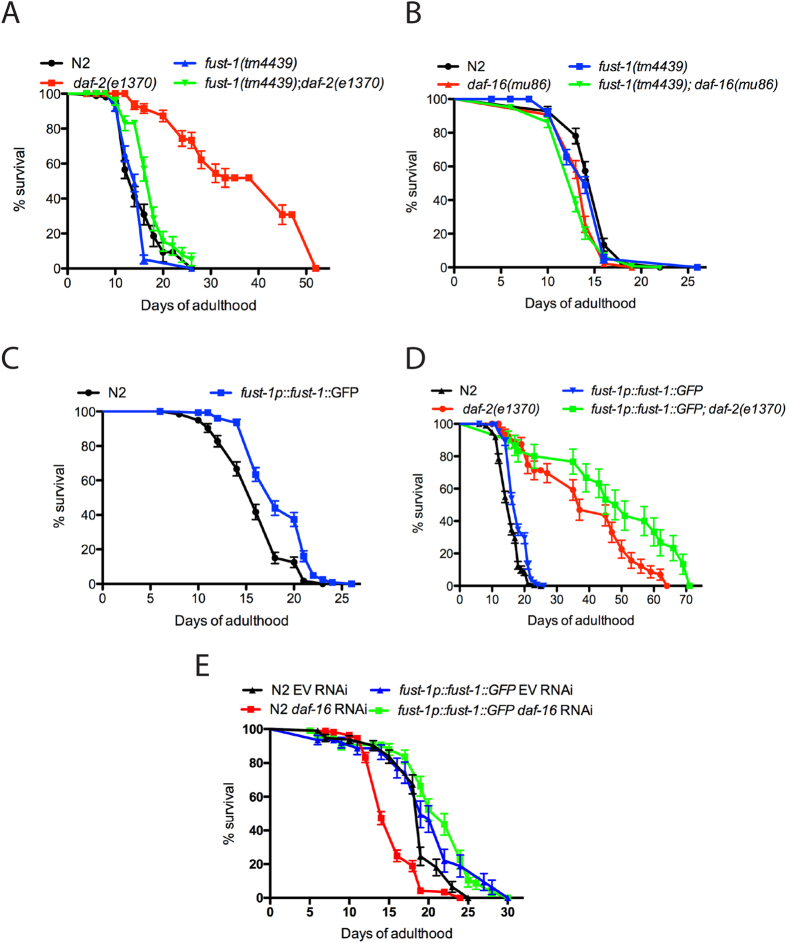
f*ust-1* functions within the IIS pathway to regulate lifespan. (**A**) Decreased expression of *fust-1* abolished the long-lived phenotype of *daf-2(e1370)* (p value < 0.005) but (**B**) has no effect on *daf-16* mutants. Overexpression of *fust-1* increased the lifespan **(C)** of N2 worms (p value < 0.0001) and (**D**) of *daf-2(e1370)* mutants (p value < 0.005) but (**E**) has no effect on *daf-16* loss of function (p value 0.4030).

Genetic signalling pathways regulating aging have been extensively studied in *C. elegans* and central to lifespan and stress response mechanisms is the insulin /IGF-like signalling pathway (IIS)^26^. DAF-2 is the sole insulin/IGF receptor in *C. elegans* and hypomorphic *daf-2* mutants are long-lived and highly resistant to environmental stress^26^. We constructed a *daf-2(e1370); fust-1(tm4439)* double mutant strain and observed that the loss of *fust-1* completely abolished the extended lifespan phenotype of *daf-2(e1370)* mutants (Fig. 2A and Supplementary Material, Table S2A). These data suggest that that *fust-1* functions within the IIS to regulate longevity.

In *C. elegans*, a crucial downstream effector of the IIS is the forkhead box O (FOXO) transcription factor encoded by *daf-16*. The long-lived phenotypes of *daf-2* mutants is completely dependent on *daf-16*^26^. *daf-16(mu86)* mutants are short-lived but *fust-1(tm4439)*; *daf-16(mu86)* double mutants have lifespan similar to *daf-16(mu86)* mutants alone (Fig. 2B and Supplementary Material, Table S2B).

We observed that although *fust-1(tm4439*) mutants have a normal lifespan at 20 °C and 25 °C (Supplementary Material, Fig. S4A,B and Table S2C,D), the overexpression of *fust-1* caused an increased lifespan compared to wild-type worms (Fig. 2C, Supplementary Material Table S2E). Additionally, the overexpression of *fust-1* had an additive effect on the lifespan of *daf-2* mutants (Fig. 2D, Supplementary Material Table S2F). However, the loss of *daf-16* did not affect the long-lived phenotype caused by the overexpression of *fust-1* (Fig. 2E). Thus our data suggest that *fust-1* is essential regulating lifespan via the IIS and that lifespan-extension is modulated by FUST-1 in a dose-dependent manner.

### FUST-1 is involved in resistance to environmental stress

**Figure 3:**
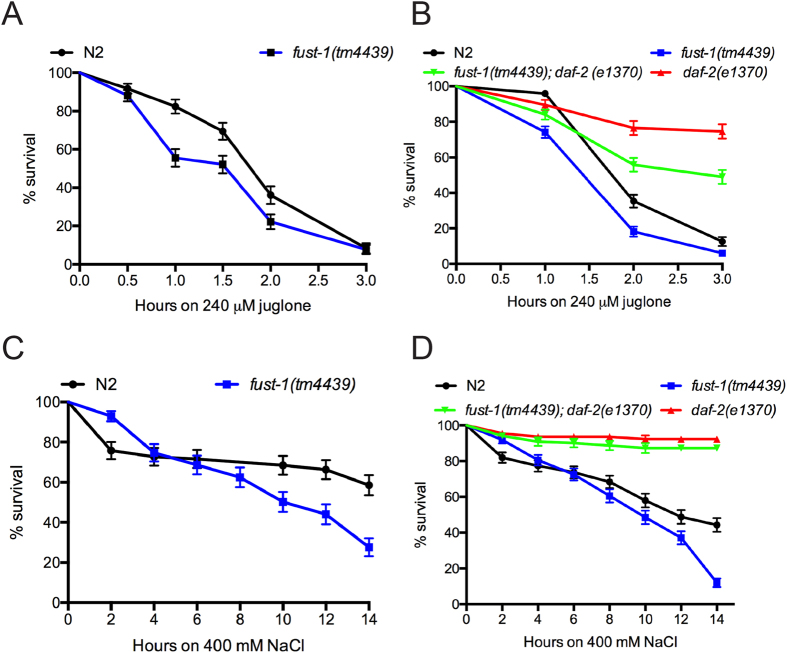
Oxidative and osmotic stress responses require *fust-1* (**A,B**) **(A)** *fust-1* mutants are more sensitive to oxidative stress induced by juglone compared to N2 controls (p value < 0.0005). **(B)** *fust-1; daf-2* mutants are more sensitive to oxidative stress than *daf-2* controls (p value < 0.0001. **(C,D) (C)** *fust-1* mutants are sensitive to osmotic stress (p value < 0.001) but the *fust-1* mutation does affect the stress sensitivity of **(D)** *daf-2(e1370)*.

Another important role of the IIS is the regulation of cellular stress responses. Worms were tested against different environmental stresses to evaluate the contribution of *fust-1* within the IIS. First, worms were exposed to juglone (5-hydroxy-1,4-naphthoquinone), a natural product that causes the production of intracellular free radical in worms causing an acute oxidative stress^27^. The *fust-1* deletion mutants were more sensitive than wild-type N2 worms (Fig. 3A) and the sensitivity was rescued by the *fust-1p::fust-1::GFP* transgene in *fust-1(tm4439*) mutants (Supplementary Material, Fig. S5A). Next we examined *daf-2(e1370); fust-1(tm4439)* double mutants and observed that these animals were more sensitive to oxidative stress than *daf-2(e1370)* mutants, but more resistant than *fust-1* mutants alone (Fig. 3B). These data suggest that the IIS pathway is only partially reliant on *fust-1* in response to oxidative stress and the action of *fust-1* in other stress response pathways could explain the intermediate phenotype observed during oxidative stress.

*fust-1* deletion mutants were then tested for their resistance to osmotic stress. *fust-1* mutants showed sensitivity to a hypertonic environment induced by NaCl (Fig. 3C) and this phenotype was partially rescued by the overexpression of *fust-1* (Supplementary Material, Fig S5B). The deletion of *fust-1* did not affect the sensitivity of *daf-2* mutants to osmotic stress (Fig. 3D) suggesting that *fust-1* is completely independent of the IIS for the regulation of osmotic stress.

Finally, worms were exposed to thermal stress. The *fust-1* deletion mutants had a normal sensitivity when submitted to 37 °C even after 14 hours (Supplementary Material, Fig. S5C). Also, the deletion of *fust-1* in *daf-2* mutants did not significantly change their resistance to this stress (Supplementary Material, Fig. S5D) suggesting that *fust-1* does not participate in responses to thermal stress.

### *fust-1* expression is regulated by the IIS

**Figure 4:**
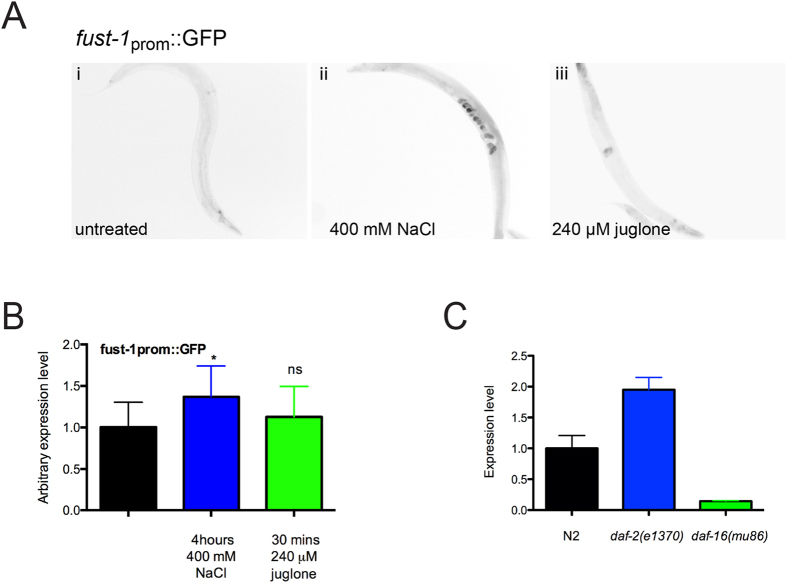
f*ust-1* expression is induced by osmotic stress and IIS. (**A**) Representative, black and white, photo-reversed images of the transgenic *fust-1p*::GFP reporter strains (control i) showing increased expression in response to osmotic (ii), but not oxidative stress (iii). (**B**) Relative quantification level of *fust-1p*::GFP under stress conditions (*p value < 0.0001, n ≥ 30 for each condition). **(C)** qRT-PCR with ΔΔCT analysis of *fust-1* expression showing an increased expression in *daf-2(e1370)* mutants and decreased expression in *daf-16(mu86)* mutants.

Genes participating in the IIS are known to have their expressions modulated under stress conditions or in IIS mutants^28^,^29^. To test if environmental stresses could induce the expression of *fust-1*, a transcriptional reporter of *fust-1* (*fust-1p::GFP*) (Supplementary Material, Fig. S6) was used to specifically evaluate the gene expression profile of *fust-1* and not the protein stability or degradation under these conditions. Worms were submitted to oxidative and osmotic stresses, the two types of stresses where *fust-1* seems to be the most involved. Osmotic but not oxidative could induce the expression of *fust-1* (Fig. 4A,B). To evaluate if *fust-1* expression is regulated by the IIS, expression level was measured in IIS mutant worms. When measured by qRT-PCR the *daf-2* mutants exhibit a two-fold higher expression level of *fust-1* than wild-type N2 (Fig. 4C). However, *daf-16* mutants exhibit a decreased expression level of *fust-1* (Fig. 4C) suggesting that *fust-1* is overexpressed when the IIS is reduced. Therefore, IIS pathway mutants have an abnormal expression of *fust-1* and osmotic stress can induce *fust-1* expression independently.

### *fust-1* involvement in neuronal integrity is independent of the IIS

**Figure 5:**
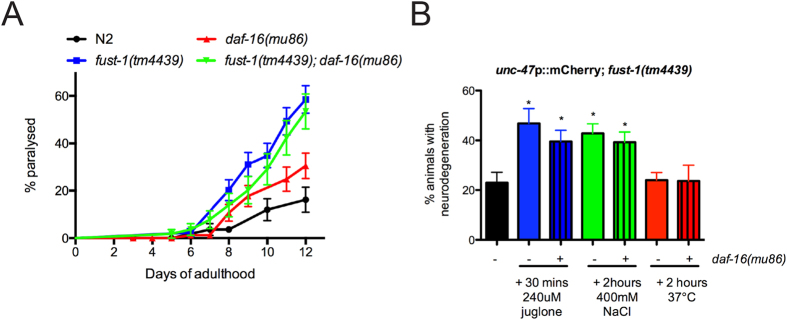
Maintenance of neuronal integrity by *fust-1* is not regulated by the IIS. **(A)** *daf-16(mu86)*; *fust-1(tm443)* mutants had rates of paralysis similar to *fust-1(tm443)* mutants alone. **(B)** Acute osmotic and oxidative stresses induce neurodegeneration in day 1 *fust-1* mutants but not thermal stress. *daf-16* mutants do not influence the % of animals with neurodegeneration (*p value < 0.05, n ≥ 60 for each conditions).

The IIS is involved in maintaining neuronal integrity^30^ and some reports have previously suggested a link between the IIS and neurodegeneration in *C. elegans*^31^,^32^. Therefore the impact of the *daf-16* mutants was tested on *fust-1* motor phenotypes. The lack of *daf-16* neither increased nor decreased the paralysis of the *fust-1(tm4439*) mutants (Fig. 5A). In previous studies, it was shown that stress sensitivity could cause neurodegeneration^14^. In order to test this hypothesis, worms were tested against oxidative, osmotic or thermal stresses and neurodegeneration of the GABAergic neurons was evaluated. Oxidative and osmotic stresses cause neurodegeneration in the *fust-1* deletion mutants but thermal stress had no significant effect (Fig. 5B), suggesting a link between the role of *fust-1* in neuronal integrity and the survival to these stresses. However, the lack of *daf-16* did not change the percentage of animals with neurodegeneration (Fig. 5B). Therefore, *fust-1* seems to be involved in neuronal integrity independently of the IIS.

## Discussion

FUS, EWS and TAF15 are RNA binding proteins that form the FET protein family. FET proteins were first identified as being involved in tumorigenesis, resulting from a DNA translocation^6^. Mutations in FET proteins have recently been linked to ALS^3^,^7^,^8^. In ALS, most mutations affecting these genes are missense mutations and cause their mislocalization from the nucleus to the cytoplasm^1^,^2^,^7^,^8^ The effect of this mislocalization and how the mutant proteins cause toxicity to motor neurons is unknown. Because of their high sequence and domain similarity, all three proteins have the same orthologue in the nematode *C. elegans*, encoded by the gene *fust-1*. Using a deletion mutant strain, we have shown here that a decrease in *fust-1* expression can cause impaired stress resistance, lifespan regulation and neuronal integrity.

Using *C. elegans*, other ALS proteins have been reported to be involved in stress and lifespan regulation. *tdp-1*, the orthologue of *TDP-43*, and *alfa-1*, the orthologue of *C9ORF72*, have been shown to impair stress response and TDP-1 was shown to be a key regulator of longevity^17^,^33^,^34^,^35^. In humans, TDP-43 localization was shown to change upon stress where wild-type TDP-43 relocalizes in stress granules^36^,^37^. In humans, FUS was also shown to participate in stress granule formation and exhibit a change in localization upon stress induction^38^,^39^. More specifically, FUS localization changes after osmotic stress and a reduction of expression of *FUS* in human cell lines induced a loss of cell viability in a hyperosmolar environment^40^. EWS and TAF-15 were also shown to translocate to stress granules in human cells and the RNA binding domains of these proteins seem essential for this phenomenon^41^,^42^. Together, these results suggest the FET proteins are involved in stress response in *C. elegans* and humans.

The *Drosophila* gene *Cabeza* is the orthologue of *FUS*, *TAF15* and *EWSR1* (from http://flybase.org/reports/FBgn0011571.html). Interestingly in *Drosophila*, *Cabeza* was also shown to be involved in lifespan, neuronal integrity, and synaptic function^10^,^11^,^13^,^43^. Synaptic dysfunction was also shown to precede neuronal loss recapitulating features observed in *C. elegans*. In a recent *FUS* knock-out model, mice with a complete loss of expression of *FUS* exhibit changes in behaviour but no motor neuron loss^44^. However, whole transcriptome analysis of spinal cord tissue of these mice has shown an increase in *EWSR1* and *TAF15* expression^44^. It is known that FUS can regulate itself though alternative splicing^45^ and it seems that it can also regulate expression of proteins with similar function such as TAF-15 and EWS-1. Therefore, using simple model organisms where compensatory mechanisms are less frequent we can reveal functions of protein families more easily and suggest here that the FET proteins act together to maintain neuron integrity and lifespan.

Using the *fust-1* deletion mutant, we have also shown that the FET protein family is involved in the IIS to maintain longevity and stress resistance. When the IIS pathway is activated, the effect of DAF-2 leads to the phosphorylation of DAF-16 that inhibits its function as a transcription factor^26^. Our lifespan and expression analysis suggested that *fust-1* is downstream of *daf-16* and that a reduction in the pathway causes an overexpression of *fust-1*. In human, IIS exerts its effect through many effectors including its many receptors, the kinases PI3K and AKT as well as FOXO transcription factors. TAF-15, EWS and FUS all contain a RNA binding domain suggestive of a role in RNA expression and metabolism. In a previous study, using UV cross-linking immunoprecipitation followed by whole transcriptome sequencing (CLIP-seq), many RNA targets of the FET protein family have been revealed. Interestingly, members of the AKT and FOXO protein family were identified as targets of wild type TAF-15, EWS and FUS proteins in human cells^5^. Using different *C. elegans* mutants, we have shown genetic interactions between the FET proteins and members of the IIS, and results from humans supports our hypothesis and suggest direct interactions with members of the IIS.

In conclusion, using *C. elegans* deletion mutants, several functions of the FET proteins have been revealed. FUST-1 is actively participating and regulated by the IIS for the maintenance of lifespan and for proper resistance to oxidative stress. Independently of the IIS, FUST-1 is involved in neuronal integrity and the osmotic stress response suggesting its participation in other stress response pathways. It is still unclear if mutations of the FET protein family causative of ALS results in a loss of function or a gain of function. However, mutations in essential tremor, another neurodegenerative disorder, were found in *FUS* and are suggestive of a loss of function mechanism^46^. Therefore, therapies targeting one of those proteins will have to be highly specific and take into account the impact on the other members of the FET family.

## Materials and Methods

### Strains and maintenance

Standard methods for culturing and handling the worms were used^47^. Worms were cultured on standard NGM media streak with OP-50 *Escherichia coli* strain at 20 °C if not specified otherwise. For a list of strains used see Supplementary Table 4. *fust-1p*::*fust*-1::GFP fosmid was obtained by the TransgenOme project^48^ and confirmed by sequencing and a transgenic strain was created by microinjection in *unc-119(ed3)* worms.

### Bioinformatic analyses

For amino acid alignment, FUST-1 protein sequence was used as query sequence (Wormbase) and compared to FUS (CCDS58454.1), EWS isoform (CCDS54513.1) and TAF15 (CCDS59279.1) as subject sequence and align using BlastP (http://blast.ncbi.nlm.nih.gov/blast/Blast.cgi?PROGRAM=blastp&PAGE_TYPE=BlastSearch&LINK_LOC=blasthome). For prediction of the functional domain of FUST-1 NetNes (http://www.cbs.dtu.dk/services/NetNES/) and Prosite (http://prosite.expasy.org/scanprosite/) were used with FUST-1 coding sequence (Wormbase). Protein alignments were done using Clustal Omega (http://www.ebi.ac.uk/Tools/msa/clustalo/) using the coding sequence mentioned above and followed by processing with BoxShade (http://www.ch.embnet.org/software/BOX_form.html).

### Paralysis assay

Worms were transferred to 5 μM FUDR plates one day after L4. Worms were scored daily for movement for 12 days. Worms were counted as paralysed if they failed to move after they were prodded on the nose. Experiments were performed at 20 °C and at least 60 worms were counted per condition. Survival curves and statistics were produced using Log-rank (Mantel-Cox) test. Standard error is shown on graphs.

### Liquid culture assay

A synchronised population was obtained using hypochlorite extraction. Worms were grown on solid media up to day 1 of adulthood. At day 1, 30 worms per well were placed in S basal with OP-50 *E. coli* (optical density 0.5) in a flat-bottom 96-well plate. Measurement was done using Microtracker (Phylumtech), at least 3 wells were done per condition. Standard error is shown on the graph.

### Neuronal integrity

To score gaps along the GABAergic neurons, day one, five and nine worms expressing the transgenic *unc-47p::mCherry* marker were selected for visualisation. To evaluate synaptic integrity, transgenic worms expressing *snb-1p::GFP* were selected. For neurodegeneration counts during stress tests, adult day one worms were transferred to NGM + 400 mM NaCl at 20 °C (osmotic stress) or normal NGM and put at 37 °C (thermal stress) for six hours or for oxidative test worms were transferred on 240 uM juglone for 30 minutes at 20 °C. For visualization, animals were immobilized in M9 with 5 mM of levamisole and mounted on slides with 2% agarose pads. For all experiments, a minimum of 100 worms was scored for all conditions. The mean and SEM were calculated and two-tailed t-tests were used for statistical analysis.

### Lifespan assays

Worms were grown on NGM and transferred on NMG + 5 μM FUDR at day 1 of adulthood. For *daf-16* RNAi lifespan RNAi clone from the ORFeome RNAi library (Open Biosystems) was used. RNAi experiments were performed at 20°C. Worms were grown on NGM enriched with 1 mM Isopropyl-b-thiogalacto-pyranoside (IPTG). Worms were counted every two days until their death. At least 100 worms were counted per strain. Survival curves and statistics were produced using Log-rank (Mantel-Cox) test. Standard error is shown on graphs.

### Stress sensitivity assay

Worms were grown on NGM until day 1 of adulthood. At day 1, worms were transferred onto 400 mM NaCl plates for osmotic stress, or 240 μM juglone for oxidative stress or onto NGM and put at 37 °C for thermal stress. Worms were counted every two hours for up to 14 hours for osmotic and thermal stress and every 30 minutes for three hours for oxidative stress. Survival curves and statistics were produced using Log-rank (Mantel-Cox) test. Standard error is shown on graphs.

### RT-PCR

Worms grown on NGM plates were collected before starvation and froze in Trizol. Total RNA was extracted according to the manufacturer protocol. 1 ug of RNA was used to produce cDNA using Vilo cDNA enzyme. Taqman probes detecting *fust-1* (probe Ce02434658_g1 Life technologies) and act-5 (probe Ce02454560_g1 Life technologies) as endogenous control were used. Expression level were calculated by converting the threshold cycle (Ct) values using the 2−ΔΔCt method^49^

## Additional Information

**How to cite this article**: Therrien, M. *et al*. FET proteins regulate lifespan and neuronal integrity. *Sci. Rep*. **6**, 25159; doi: 10.1038/srep25159 (2016).

## Supplementary Material

Supplementary Information
