# Supplementary material for: FET proteins regulate lifespan and neuronal integrity
Source: Sci Rep. 2016 Apr 27;6:25159. doi: 10.1038/srep25159 (PMC4846834; doi:10.1038/srep25159)
Supplement: Supplementary Information [file srep25159-s1.pdf]

Supplementary Material

**Title:** FET proteins regulate lifespan and neuronal integrity

**Authors:** Martine Therrien[1,2], Guy A. Rouleau[3,4], Patrick A. Dion[3,4] and J. Alex Parker[1,5]*

**Affiliations:** [1] CHUM Research Center, Montreal, H2X 3H8, Canada

[2] Pathology and Cell biology department, University of Montreal, Montreal, H3T 1J4, Canada.

[3] Neurology and Neurosurgery department, McGill University, Montreal, H3A 0G4, Canada.

[4] Montreal Neurological Hospital, Montreal, H3A 2B4, Canada.

[5] Department of Neuroscience, University of Montreal, Montreal, H3T 1J4, Canada.

***Corresponding Author:** ja.parker@umontreal.ca

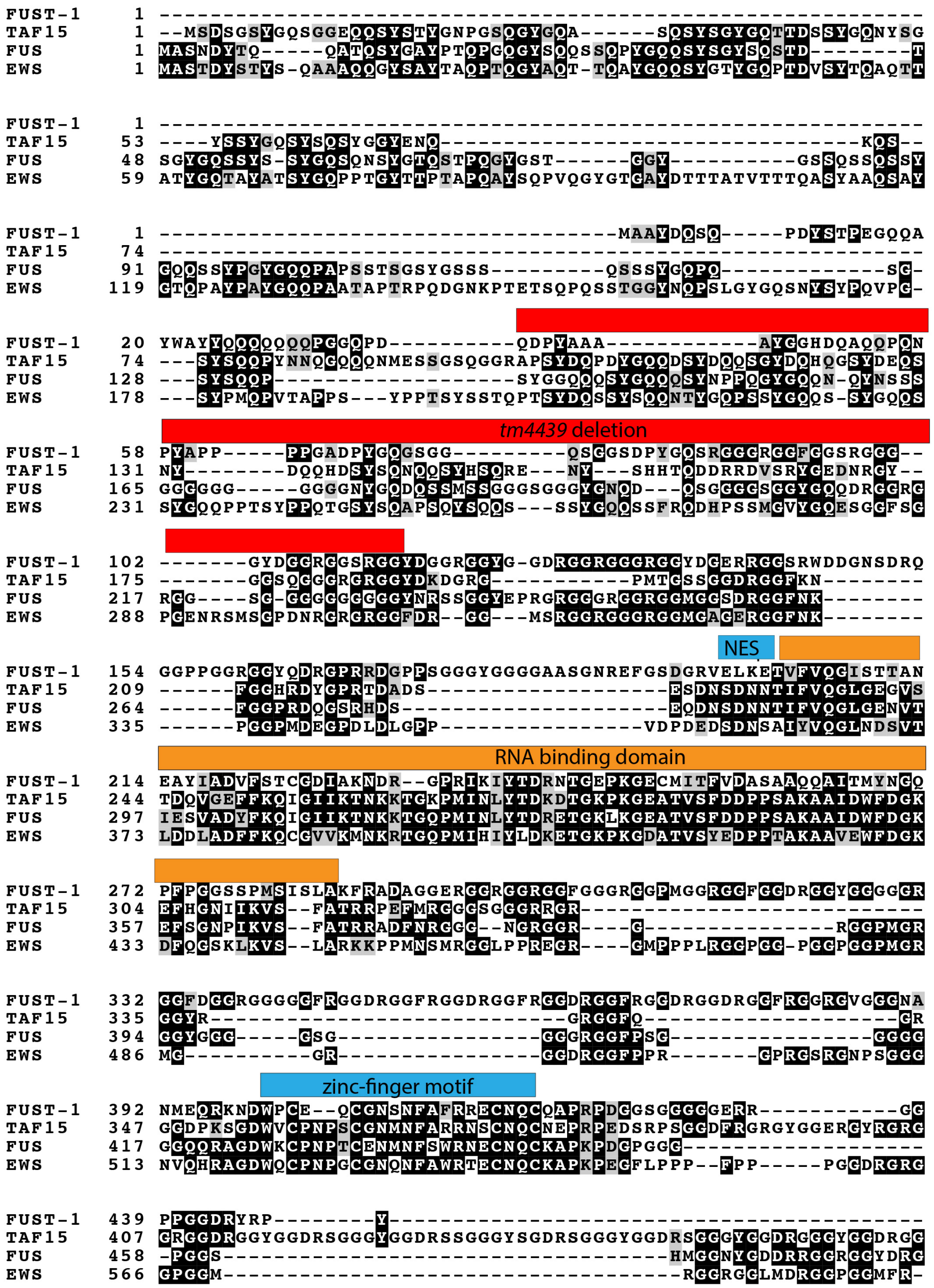


**Supplementary Figure 1**. FUST-1 is the ortholog of FUS, EWSR1 and TAF15. Protein sequence alignment of *C. elegans* FUST-1 and human EWS, TAF-15 and FUS proteins showing that the nuclear export signal (NES), the RNA binding domain and the zinc-finger motif are highly conserved between the *C. elegans* and human proteins.

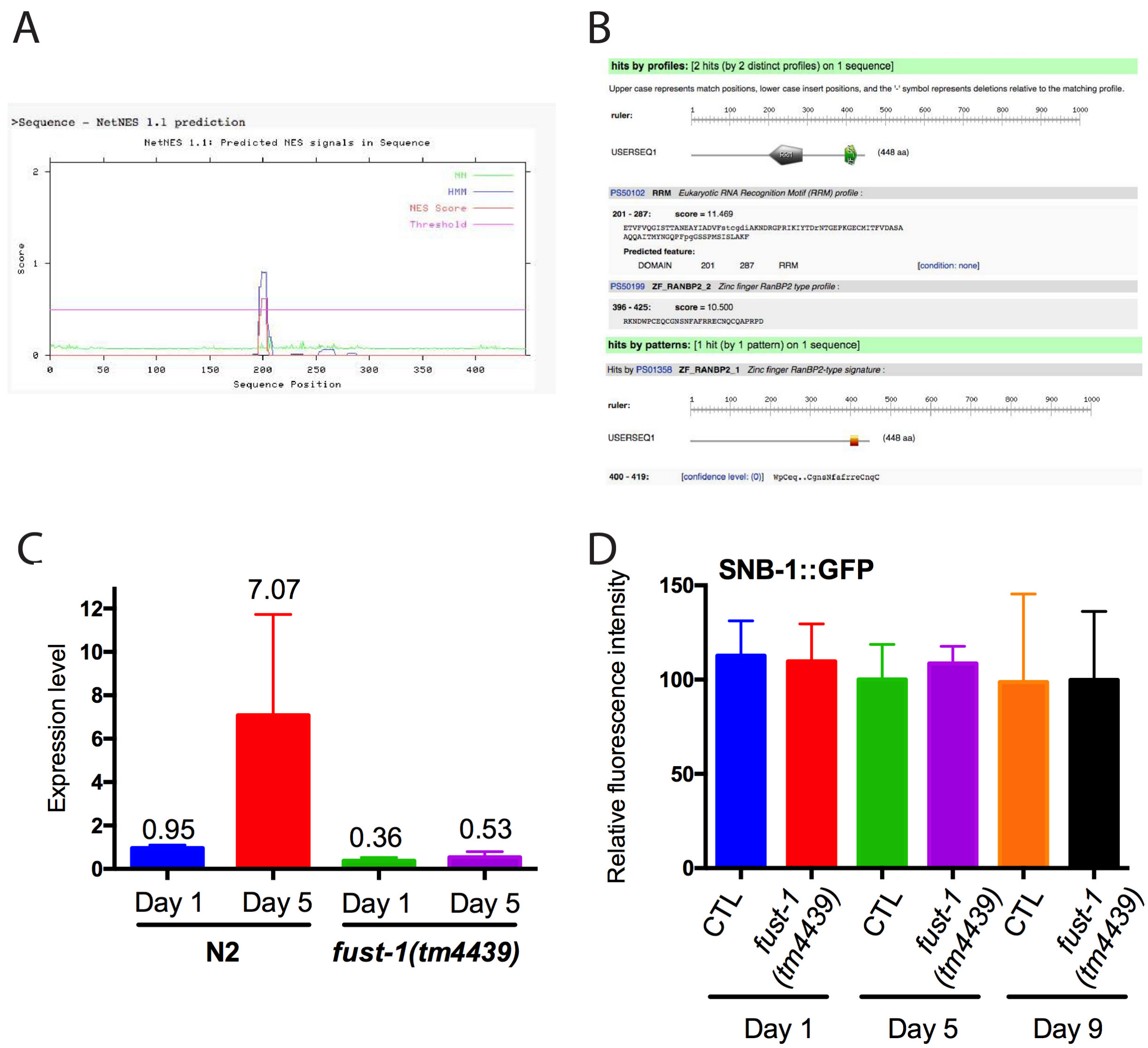


**Supplementary Figure 2**. FUST-1 has the functional domain of human FUS, EWSR1 and TAF15. **(A)** NetNES prediction showing the presence of a potential NES at amino acid 200 of FUST-1. **(B)** Prosite prediction of FUST-1 showing the presence of an RNA-recognition motif and a zinc-finger motif. **(C)** qRT-PCR with ΔΔCT analysis of *fust-1* expression is increased during adulthood in wild-type animals while in *fust-1(tm4439)* mutant the expression level is least 50% lower than in wild-type N2 worms. (D) Fluorescence intensity of SNB-1::GFP in wild-type and *fust-1(tm4439)* mutant. No change is observed at Days 1, 5 and 9 of adulthood.

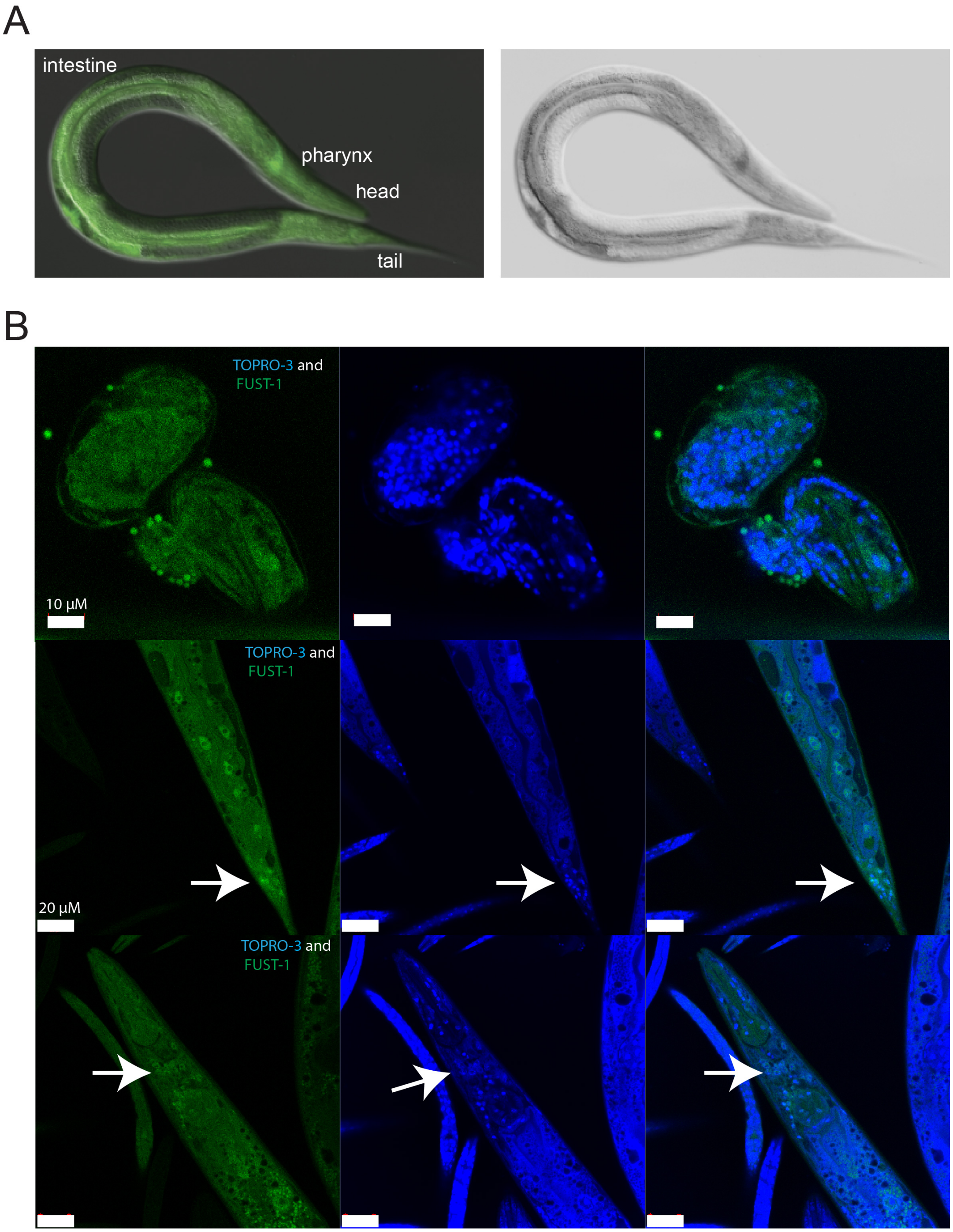


**Supplementary Figure 3.** FUST-1 overexpression strain **(A)** Picture of *fust-1p*::*fust-1*::GFP strain showing expression of *fust-1* in head, pharynx, intestine and tail of the adult animal. Left panel is showing GFP image merged with DIC image and right image is black and white inversion of the same image. **(B)** FUST-1 (green) is found in the nucleus (blue) of some cells in the pharynx and tail of adult animals (arrow) but not in the embryo.

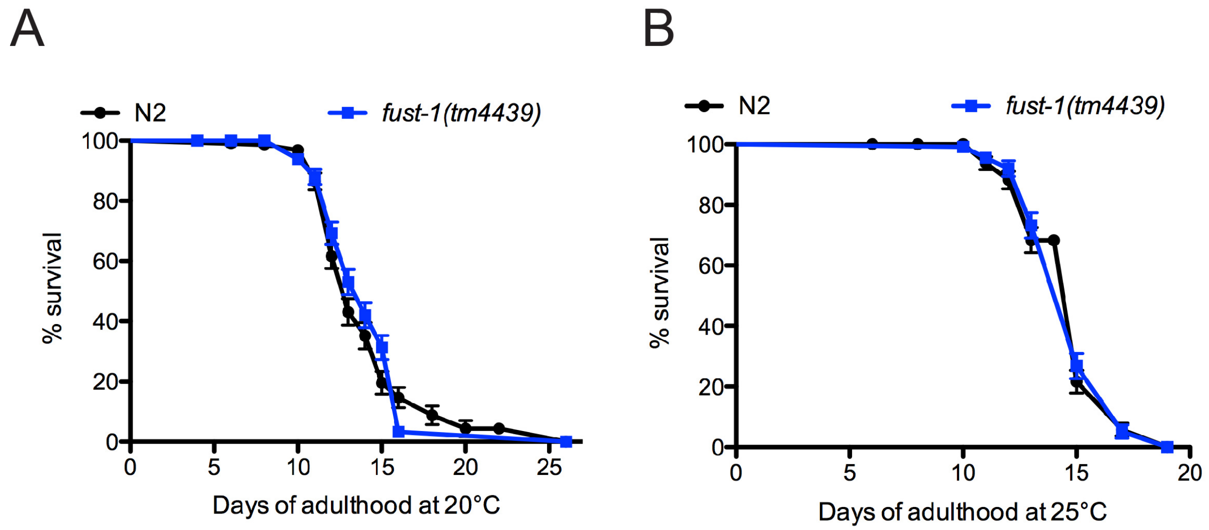


**Supplementary Figure 4.** Loss of *fust-1* does not affect lifespan. *fust-1(tm4439)* mutants had lifespans similar to N2 controls when grown at either **(A)** 20°C or **(B)** 25°C.

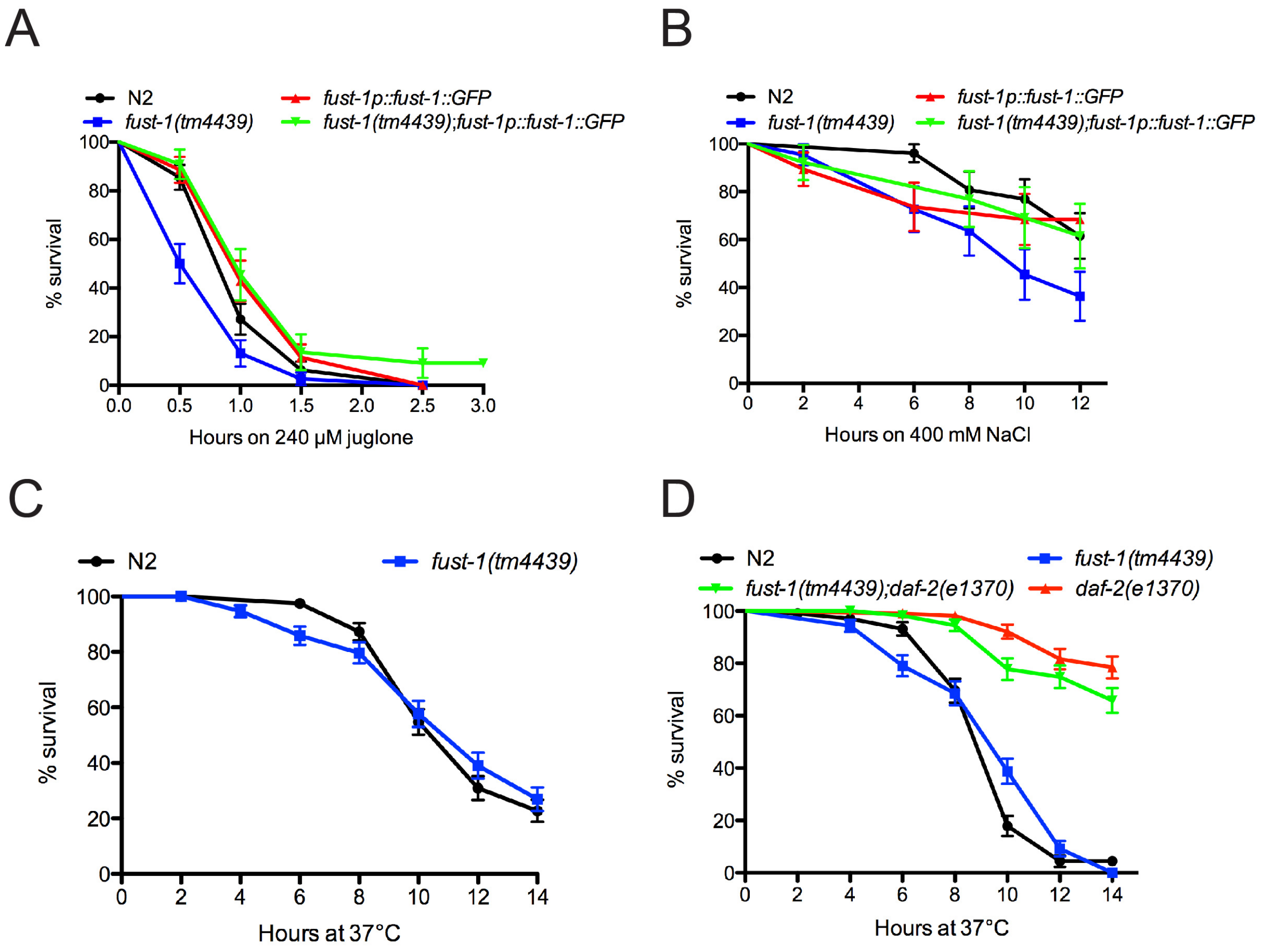


**Supplementary Figure 5.** Overexpression of *fust-1* rescues stress sensitivity of *fust-1(tm4439)* mutants. Overexpression of *fust-1* **(A)** rescues *fust-1(tm4439)* sensitivity to juglone (p value <0.001) and **(B)** partially rescues sensitivity to osmotic stress (p value< 0.16). **(C-D)** Decreased expression of *fust-1* does not affect response to thermal stress of wild-type and *daf-2* mutants.

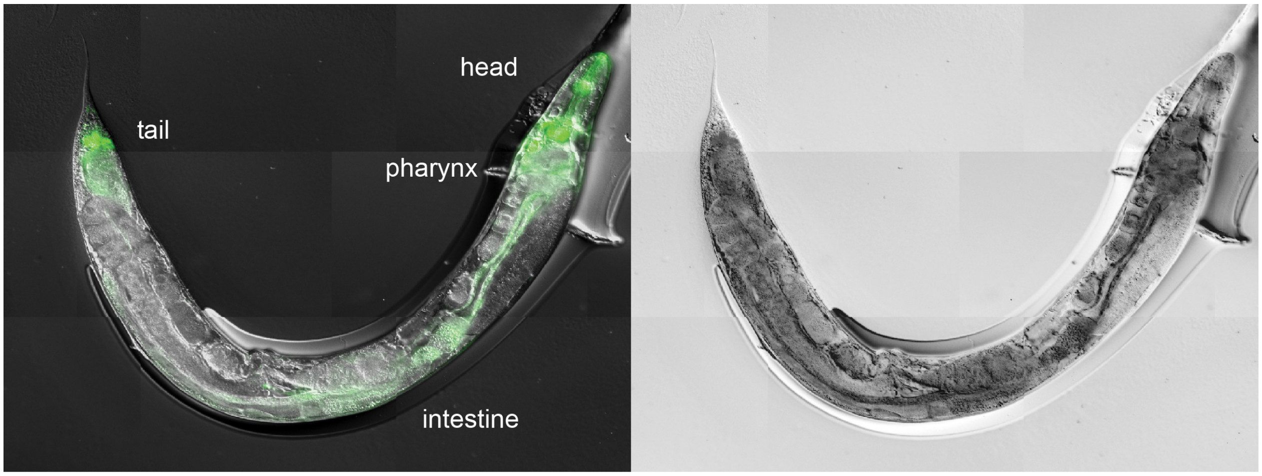


**Supplementary Figure 6.** Representative pictures of *fust-1p*::GFP worms. The left panel is a picture of a *fust-1p*::GFP transgenic showing expression of *fust-1* in head, pharynx, intestine and tail of the adult animal. The left panel shows GFP and DIC images, and right panel is the black and white inversion of the image in the left panel.

**Supplementary Table 1**

A) paralysis on solid media of *fust-1(tm4439)*

| | % paralysed at day 12 | P value vs N2 |
|---|---|---|
| N2 | 13% | --- |
| *fust-1(tm4439)* | 66% | <0.0001 |

B)paralysis in liquid culture of *fust-1(tm4439)*

| | P value vs N2 |
|---|---|
| N2 | --- |
| *fust-1(tm4439)* | <0.0001 |

C) *fust-1prom*::FUST-1::GFP rescue *fust-1(tm4439*) motor phenotype

| | % paralysed at day 12 | P value vs N2 | P value vs *fust-1 (tm4439)* |
|---|---|---|---|
| N2 | 16% | --- | 0.0010 |
| *fust-1(tm4439)* | 43% | 0.0010 | ----- |
| *fust-1p::fust-1::GFP* | 21% | 0.5907 | <0.0001 |
| *fust-1(tm4439); fust-1p::fust-1::GFP* | 22% | 0.6629 | 0.0006 |

**Supplementary Table 2**

A) *fust-1(tm4439)* decreased long-lived phenotype of *daf-2(e1370)* mutants

| | Median survival | P value vs N2 | P value vs *fust-1 (tm4439)* |
|---|---|---|---|
| N2 | 14 days | --- | 0.4206 |
| *fust-1(tm4439)* | 14 days | 0.4206 | --- |
| *daf-2(e1370)* | 45 days | <0.0001 | <0.0001 |
| *fust-1(tm4439);daf-2(e1370)* | 18 days | 0.003 | <0.0001 |

B) *fust-1(tm4439)* does not affect *daf-16* mutants lifespan

| | Median survival | P value vs N2 | P value vs *fust-1 (tm4439)* |
|---|---|---|---|
| N2 | 16 days | --- | 0.0761 |
| *fust-1(tm4439)* | 14 days | 0.0761 | ----- |
| *daf-16(mu86)* | 14 days | <0.0001 | 0.0500 |
| *fust-1(tm4439);daf 16(mu86)* | 13 days | <0.0001 | 0.0072 |

C) *fust-1(tm4439)* has no effect on lifespan at 20°C

| At 20°C | Median survival | P value vs N2 |
|---|---|---|
| N2 | 13 days | --- |
| *fust-1(tm4439)* | 14 days | 0.5728 |

D) *fust-1(tm4439)* has no effect on lifespan at 25°C

| At 25°C | Median survival | P value vs N2 |
|---|---|---|
| N2 | 15 days | --- |
| *fust-1(tm4439)* | 15 days | 0.3910 |

E) *fust*-1 overexpression increases lifespan

| At 20°C | Median survival | P value vs N2 |
|---|---|---|
| N2 | 16 days | --- |
| *fust-1p::fust-1::*GFP | 18 days | <0.0001 |

F) *fust-1* overexpression increases *daf-2(e1370)* lifespan

| | Median survival | P value vs N2 | P value vs *fust-1prom*::FUST-1::GFP |
|---|---|---|---|
| N2 | 16 days | --- | <0.0001 |
| *fust-1p::fust-1::GFP* | 18 days | <0.0001 | ----- |
| *daf2(e1370)* | 37 days | <0.0001 | <0.0001 |
| *fust-1p::fust-1::GFP;daf-2(e1370)* | 49.5 days | <0.0001 | <0.0001 |

| | Median survival | P value vs N2 | P value vs *fust-1prom*::FUST-1::GFP |
|---|---|---|---|
| N2 EV RNAi | 19 days | --- | 0.0490 |
| *fust-1p::fust-1::GFP* EV RNAi | 19 days | 0.0490 | ----- |
| *N2 daf-16* RNAi | 14 days | <0.0001 | <0.0001 |
| *fust-1p::fust-1::GFP daf-16* RNAi | 22 days | <0.0001 | 0.4030 |

**Supplementary Table 3 –*daf-16* does not affect paralysis rate of *fust-1(tm4439)***

| | % paralysed at day 12 | P value vs N2 | P value vs *fust-1 (tm4439)* |
|---|---|---|---|
| N2 | 16% | --- | <0.0001 |
| *fust-1(tm4439)* | 58% | <0.0001 | ----- |
| *daf-16(mu86)* | 30% | 0.0708 | 0.0008 |
| *fust-1(tm4439); daf-16(mu86)* | 53% | <0.0001 | 0.5059 |

**Supplementary Table 4 - List of strains**

| Strain | source | genotype |
|---|---|---|
| N2 | CGC | |
| *FX4439* | National Bioresource Project of Japan | *fust-1(tm4439)* |
| *ufIs34* | M Francis lab | *ufIs34* (unc-47::mCherry) |
| CZ333 | CGC | unc25p::snb-1::GFP |
| CB307 | CGC | *unc-47(e307)* |
| CB246 | CGC | *unc-64(e246)* |
| CB1370 | CGC | *daf-2(e1370)* |
| CF1038 | CGC | *daf-16(mu86)* |
| XQ 307 | JA. Parker lab | *unc-119(ed3);xqEx307* (fust-1p::fust-1::GFP) |
| BC10929 | CGC | *dpy-5(e907)*I;*sEx10929*(rCes C27H5.3::GFP + pCeh361) |
